# Elevated Levels of IL-27 Are Associated with Disease Activity in Patients with Crohn's Disease

**DOI:** 10.1155/2021/5527627

**Published:** 2021-10-26

**Authors:** Xiufang Cui, Chunhua Jiao, Di Wang, Ziping Ye, Jingjing Ma, Nana Tang, Hongjie Zhang

**Affiliations:** ^1^Department of Gastroenterology, First Affiliated Hospital of Nanjing Medical University, Nanjing, Jiangsu 210029, China; ^2^Department of Gastroenterology, Nanjing First Hospital, Nanjing Medical University, Nanjing, Jiangsu Province, China

## Abstract

Immune disorders play an important role in the pathogenesis of Crohn's disease (CD). Notably, the increased immune response of Th1 cells and related cytokines is associated with the onset of CD. IL-27 is a newly discovered IL-12-related cytokine, but its expression and clinical significance in CD patients are still controversial. This study is aimed at evaluating the serum levels of IL-27 in CD patients and analyzing their clinical significance. The results indicated that serum levels of IL-27 in CD patients were significantly higher than those in control subjects (median (interquartile range (IQR)): 110.0 (95.0, 145.0) vs. 85.0 (80.0, 95.0) pg/ml, *P* < 0.001). Furthermore, the IL-27 levels significantly increased in CD patients at the active stage compared with CD patients in remission (CDR) (127.5 (100.0, 150.0) vs. 90 (80.0, 110.0) pg/ml, *P* < 0.001). However, there was no difference in IL-27 levels between CDR and control subjects. The levels of IL-27 were positively correlated with Crohn's disease activity index (CDAI), C-reactive protein (CRP), erythrocyte sedimentation rate (ESR), fecal calprotectin (FC), and Simple Endoscopic Score for Crohn's Disease (SES-CD) and negatively correlated with hemoglobin (Hb) and serum albumin (ALB). IL-27 combined with CRP favored the prediction of CD activity (area under the curve (AUC): 0.88). Additionally, the proportions of Th17 and Th1 cells in peripheral blood were higher in CD patients than in control subjects. Active CD patients exhibited significantly higher proportions of Th17 and Th1 cells than those in remission. Moreover, correlation analysis indicated that the serum levels of IL-27 were positively associated with the frequency of Th17 cells in CD patients (*r* = 0.519, *P* = 0.013) but not associated with the frequency of Th1 cells in CD patients. IL-27 is positively associated with multiple inflammation indicators and may exert a proinflammatory profile by regulating Th17 cell differentiation in the development of Crohn's disease. In the future, IL-27 combined with CRP is expected to become an important biological marker of CD activity.

## 1. Introduction

Crohn's disease (CD) is a chronic nonspecific inflammatory disease of the gastrointestinal tract with unknown pathogenesis [1]. At present, emerging studies confirm that genetic, environmental, microorganism, and immune disorders are involved in the pathogenesis of CD. Studies have shown that the cellular immune response of the intestinal mucosa to unknown pathogens is uncontrolled or inappropriately upregulated, which plays an important role in the triggering and persistence of the disease. As a result of this uncontrolled immune response, immune cells infiltrate into the lamina propria and peripheral circulation, leading to the release of a large number of inflammatory cytokines and further aggravating the progression of the disease. Recently, it has been shown that the response of T helper cells is associated with inflammation by releasing proinflammatory cytokines, including interleukin- (IL-) 2, IL-17, IL-1*β*, IL-12, and TNF-*α* [2]. These cytokines are involved in the progression of diseases by regulating the inflammatory process. Among these, Th17 cells play a vital role in autoimmune diseases and protective host immunity [3]. Mechanistically, the frequencies of Th17 cells and related cytokines were increased in peripheral blood mononuclear cells (PBMCs) and the intestinal mucosa of CD patients [4].

T cells can differentiate into different effector T cells upon antigen stimulation. This process is involved in cytokine release from other cells, especially non-T cells such as macrophages, dendritic cells, and B cells. The newly discovered heterodimeric cytokine IL-27 consists of the p28 protein, which is related to the p35 subunit of IL-12, and Epstein–Barr virus-induced gene 3 (EBI3), an IL-12p40-related protein [5]. IL-27 is categorized as a type 1 cytokine defined by shared structural motifs, such as the four-helix bundle and the hematopoietin receptor domain, as well as similar signaling properties among the ligands and receptors that are common to other members of the IL-12 family, including IL-6 and IL-23 [6, 7]. IL-27 mediates intracellular signals through heterodimeric receptors (IL-27R) composed of gp130 and WSX-1 [8]. IL-27R is expressed on a variety of immune cells. It stimulates the rapid expansion of T cells and cooperates with IL-12 to trigger IFN-*γ* secretion. Studies have confirmed that IL-27 has both proinflammatory effects and anti-inflammatory effects, showing a dual role in the process of inflammation. IL-27 mediates Th1 differentiation and IFN secretion by inducing T-bet expression in a STAT1-dependent manner [9]. A recent study from Visperas et al. demonstrated that IL-27 played a key role in optimizing cell differentiation and increasing Th17 cytokine secretion, which further triggered an inflammatory response [10]. In addition, IL-27 has the ability to inhibit the differentiation of Foxp3+ regulatory T (Treg) cells [11, 12]. Th1 and Th17 cells are involved in the pathogenesis of autoimmune diseases, including CD, atherosclerosis, and liver injury [13]. Previous research indicated that elevated IL-27 levels were positively correlated with Th17 cells in patients with liver injury [14]. The above research results emphasized the proinflammatory effect of IL-27. However, some previous studies revealed that IL-27 also exerts a complex function, such as anti-inflammatory properties [15, 16]. Delivery of IL-27 to the model of experimental autoimmune encephalomyelitis (EAE) can relieve CNS inflammation by inhibiting Th17 and Th1 cells [17]. Villarino et al. [18] reported that IL-27 suppressed IL-2 release from CD4^+^ T cells.

In terms of intestinal inflammation, the function of IL-27 is also controversial. Transferring of IL-27R*α*^−/−^ CD4^+^ T cells to immunodeficiency mice relieved colitis by increasing inducible Treg cells [19]. In vitro experiments showed that IL-27 increased MHC and TLR4 expression, subsequently causing increased release of IL-6 and IL-1*β* in human monocytes [20]. Additionally, the IL-27 mRNA level in mucosal tissue biopsy samples of active IBD patients was higher than that of the control group. Increased IL-27 expression in IBD patients supports its proinflammatory effect [21]. In contrast, Sasaoka et al. [22] showed that IL-27 inhibits intestinal inflammation by regulating Th17 differentiation.

Therefore, the role of IL-27 in the development of CD remains unclear. We conducted this study to measure the serum levels of IL-27 in CD patients and analyzed the association between IL-27 levels and disease activity. Furthermore, we examined the percentage of Th17 and Th1 cells in peripheral blood and analyzed the relationship between the levels of IL-27 and the percentage of T cells in peripheral blood.

## 2. Materials and Methods

### 2.1. Ethics Approval

The design protocol was approved by the Ethics Committee of the First Affiliated Hospital of Nanjing Medical University. Written informed consent forms in Chinese were obtained from all participants.

### 2.2. Subjects

A total of 60 CD patients (47 males and 13 females, median age 27) were enrolled in this observational study, and 32 control subjects (20 males and 12 females, median age 30) were selected as controls after matching age and gender to the patient cohort between June 2019 and October 2020 from the Department of Gastroenterology in the First Affiliated Hospital of Nanjing Medical University. CD diagnosis was determined by combining clinical symptoms, endoscopy findings, and histopathology compatible with the European Crohn's and Colitis Organization guidelines [1]. The control individuals (*n* = 32) were selected from the healthcare center by matching age and sex and underwent endoscopy examination. No evidence of colitis or neoplasia was found according to the endoscopic and histological manifestations. The disease activity of CD was evaluated by Crohn's disease activity index (CDAI) and Simple Endoscopic Score for Crohn's Disease (SES-CD). The clinical course of CD was defined as clinical remission and active according to CDAI (CDAI≦150 as clinical remission; CDAI ≥ 150 as clinical active) [23]. According to the SES-CD scores, the CD patients were divided into endoscopic remission (SES-CD score≦2 points) and active groups (SES-CD score > 2) [24]. The relevant information of all enrolled CD patients was collected from electronic medical records, including age, sex, onset age of disease, location of lesions, disease course, previous and current medical treatment, and history of bowel resection. We recorded the values of serum inflammatory indicators, including C-reactive protein (CRP), erythrocyte sedimentation rate (ESR), platelet (PLT) counts, nutritional indicators, including hemoglobin (Hb) and serum albumin (ALB), and fecal calprotectin (FC).

### 2.3. Flow Cytometry

Fluorescently labeled antibodies or isotype-matched controls, including FITC-conjugated anti-CD8, APC-conjugated CD3, PE-conjugated anti-IL-17A, and PE-conjugated anti-IFN-*γ*, were purchased from BioLegend Company (San Diego, CA, USA). Briefly, fresh heparinized peripheral blood was incubated for 5 hours at 37°C in a humidified 5% CO_2_ incubator by stimulation with phorbol 12-myristate 13-acetate (PMA, 50 ng/ml, Sigma) and ionomycin (1 *μ*M, Sigma) in the RPMI 1640 medium supplemented with 10% fetal bovine serum (FBS). GolgiPlug (BD Biosciences) was subsequently added during the last 3 hours of incubation. Collected cells were washed three times with PBS and then surface-stained with anti-CD8 and anti-CD3 antibodies at 4°C for 30 minutes. Then, the cells were fixed and permeabilized using a FIX&PERM Kit (MultiSciences Biotech Co., Ltd.) according to the manufacturer's instructions, followed by staining with PE-conjugated anti-IL-17A or PE-conjugated IFN-*γ*. Data were analyzed using FlowJo software (TreeStar, Ashland, OR, USA).

### 2.4. Enzyme-Linked Immunosorbent Assay

The serum of blood samples was collected from each subject, and the concentration of IL-27 was quantified by using a LEGEND MAX™ Human IL-27 ELISA kit (BioLegend, San Diego, CA). The concentration of IL-23 was detected by using a LEGEND MAX™ Human IL-23 ELISA kit (BioLegend, San Diego, CA). The protocol was performed according to the manufacturer's protocols. The sensitivity of the ELISA kits was 11 pg/ml for IL-27 and 2.45 pg/ml for IL-23.

### 2.5. Statistical Analysis

All the data were expressed as the median (interquartile range (IQR)) and analyzed using SPSS 25.0 software (IBM Company, Armonk, NY). The differences among groups were analyzed by using the Kruskal–Wallis test and Mann–Whitney*U*test. Spearman's rank correlation test was used to evaluate correlation analysis. The receiver operating characteristic (ROC) curves and the areas under the ROC curves (AUCs) were analyzed by using the *Z*-statistic. All graphs were drawn using GraphPad software 8.0 (GraphPad Inc., San Diego, CA). A two-sided *P* value lower than 0.05 was considered to be statistically significant.

## 3. Results

### 3.1. Clinical Characteristics of CD Patients and Control Subjects

We enrolled a total of 60 CD patients (47 males and 13 females, median age 27) in this observational study. Thirty-two control subjects (20 males and 12 females, median age 30) were selected and were sex- and age-matched to the patient cohort between June 2019 and October 2020 from the Department of Gastroenterology in the First Affiliated Hospital of Nanjing Medical University. There were 38 CD patients with active disease and 22 in remission. No significant differences were found in sex or age between the CD and control groups (Table 1).

### 3.2. Comparison of IL-27 Levels in Peripheral Serum of CD Patients and Control Subjects

**Figure 1:**
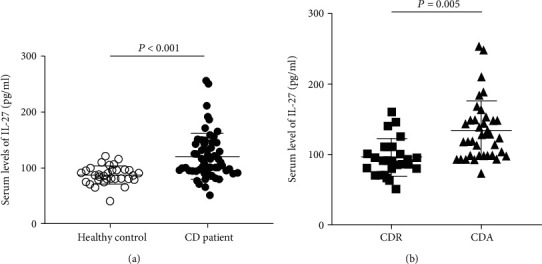
Comparison of serum IL-27 levels in different groups. (a) Serum IL-27 levels in control subjects and Crohn's disease patients. (b) The histogram shows the serum IL-27 levels in CD patients in remission (CDR) and CD patients in activity (CDA). The differences between the two groups were determined by the nonparametric Mann–Whitney rank test.

IL-27 levels in the serum of CD patients and control subjects were assessed by ELISA, and the results are shown in Figure 1(a). The median serum IL-27 levels were significantly elevated in all CD patients compared to the control subjects (median (IQR), CD vs. HC: 110.0 (95.0, 145.0) vs. 85.0 (80.0, 95.0) pg/ml, *P* < 0.001). Furthermore, we divided the CD patients into two groups according to CDAI as follows: CD patients in activity (CDA) and CD patients in remission (CDR) and compared the serum IL-27 levels between the two groups. The results indicated that serum IL-27 levels were significantly higher in the CDA groups than in the CDR groups (median (IQR): 127.5 (100.0, 150.0) vs. 90 (80.0, 110.0) pg/ml, *P* = 0.005, Figure 1(b)). These results showed that the higher the serum level of IL-27 in CD patients, the more severe the disease activity. Furthermore, the levels of IL-23 were also determined using ELISA, and the results showed that the levels of IL-23 in the serum of CD patients were slightly higher than those of the control group. However, there were no statistically significant differences in serum IL-23 levels between the groups (*P* = 0.162). Therefore, this study further explored the relationship between elevated IL-27 and CD activity.

### 3.3. The Association between Inflammatory Activity Biomarkers and Serum Levels of IL-27

**Figure 2:**
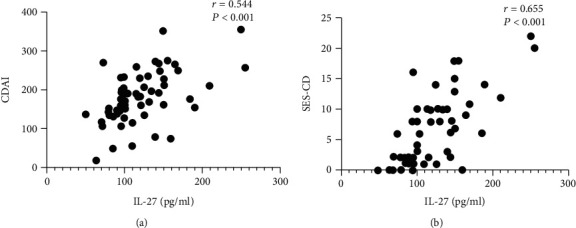
Correlation analysis between serum IL-27 levels and clinical and endoscopic activity scores in all CD patients (*n* = 60). (a) Correlation of serum IL-27 levels with Crohn's disease activity index (CDAI). (b) Correlation of serum IL-27 levels with Simple Endoscopic Score for Crohn's Disease (SES-CD). Spearman's rank correlation test was used to assess the correlations.

**Figure 3:**
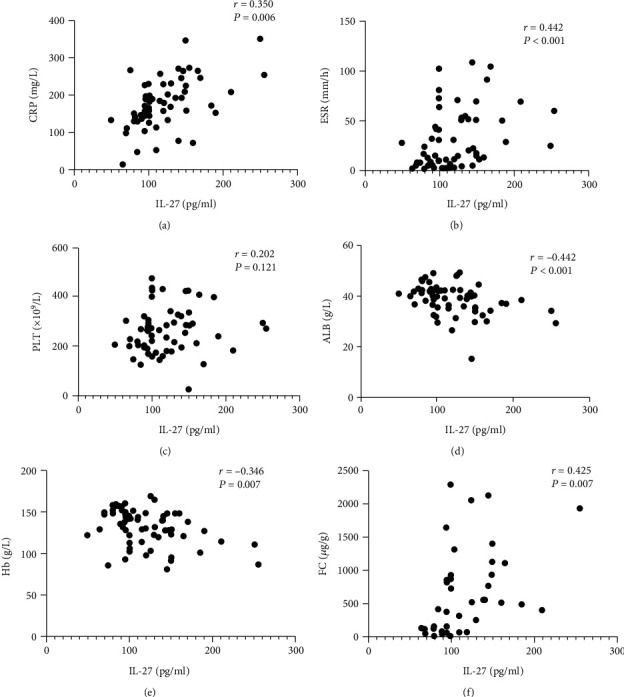
Correlation analysis between serum IL-27 levels and serum and fecal activity parameters in all CD patients (*n* = 60). (a) Correlation of serum IL-27 levels with C-reactive protein (CRP). (b) Correlation of serum IL-27 levels with erythrocyte sedimentation rate (ESR). (c) Correlation of serum IL-27 levels with platelet (PLT) counts. (d, e) Correlation of serum IL-27 levels with serum albumin (ALB) and hemoglobin (Hb). (f) Correlation of serum IL-27 levels with fecal calprotectin (FC). Spearman's rank correlation test was used to assess the correlations between the different parameters.

To assess the relationship between IL-27 levels and disease activity, we first analyzed the correlation between serum IL-27 levels and CDAI, which reflected clinical activity. As shown in Figure 2(a), the results showed that serum IL-27 levels had higher correlations with CDAI (*r* = 0.544, *P* < 0.001). We subsequently analyzed the correlation between serum levels of IL-27 and serum inflammatory biomarkers, as well as fecal calprotectin (FC), by using Spearman's rank correlation methods. As shown in Figure 3, CRP (*r* = 0.350, *P* = 0.006), ESR (*r* = 0.442, *P* < 0.001), and FC (*r* = 0.425, *P* = 0.007) were positively correlated with serum levels of IL-27 but negatively correlated with ALB (*r* = −0.442, *P* < 0.001) and Hb (*r* = −0.346, *P* = 0.007). In addition, the correlation between IL-27 and SES-CD was also analyzed, and the results showed that serum IL-27 levels had higher correlations with SES-CD (*r* = 0.655, *P* < 0.001, Figure 2(b)).

### 3.4. Predictive Values of IL-27 in CD Activity

**Figure 4:**
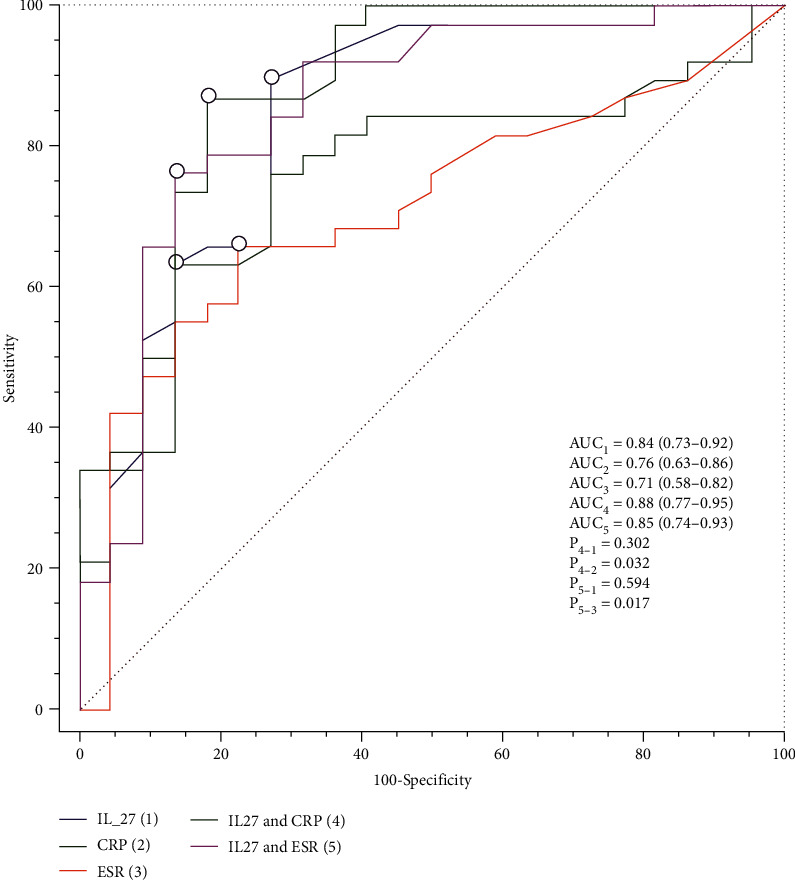
Discriminatory power of IL-27, CRP, ESR, IL-27+CRP, and IL-27+ESR for the disease activity of CD patients by using receiver operating characteristic (ROC) curves. Areas under the ROC curves (AUCs) were analyzed by using the *Z*-statistic. CRP: C-reactive protein; ESR: erythrocyte sedimentation rate; CD: Crohn's disease.

To determine the predictive value of IL-27 for disease activity in CD patients, ROC curves were drawn in Figure 4, and the significant differences in AUCs for different biomarkers were analyzed by the *Z*-statistic. As shown in Figure 4, the AUC of serum IL-27 (AUC1 = 0.84) had an increasing trend compared to that of CRP (AUC2 = 0.76) and ESR (AUC1 = 0.71), but there was no difference (all *P* values for *Z*‐statistics > 0.05). Therefore, we calculated the AUC of IL-27 combined with CRP and IL-27 combined with ESR to predict disease activity. Based on the *Z*-statistic results, we found that the AUC of IL-27 combined with CRP was 0.88 (AUC4 = 0.88), and the AUC of IL-27 combined with ESR was 0.85 (AUC5 = 0.85). The AUC of IL-27 combined with CRP in predicting disease activity was the highest, which was higher than that of CRP alone (*P* value for *Z*‐statistic = 0.032) and was not better than that of IL-27 alone (*P* value for *Z*‐statistic = 0.302).

### 3.5. The Proportion of Th17 and Th1 Cells in the Peripheral Blood of CD Patients and the Relationship with Serum Levels of IL-27

**Figure 5:**
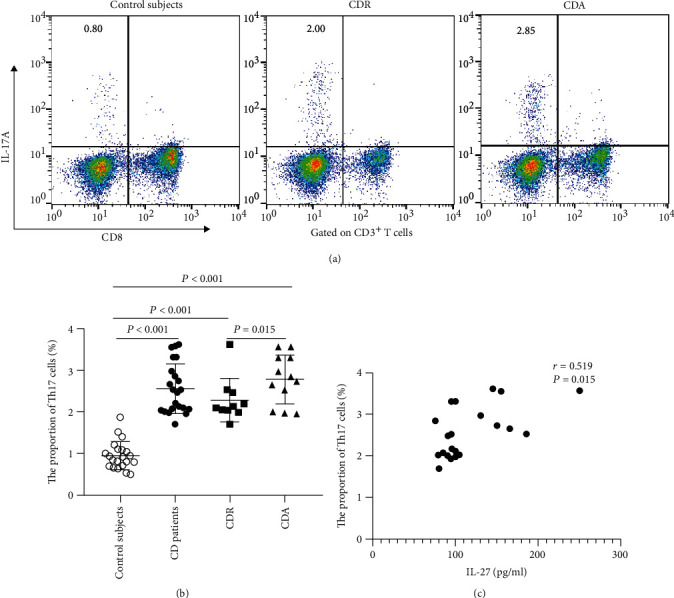
Circulating Th17 cells in healthy control subjects, all CD patients, CDR patients, and CDA patients and their relationship with serum IL-27 levels. (a) Representative dot plots of IL-17A expression in peripheral CD4^+^ T cells (gate CD3^+^CD8^−^ T cells) of HC subjects, all CD patients, CDR, and CDA. (b) Scatter plot showing the proportion of circulating Th17 cells and the results of statistical analysis by using the nonparametric Mann–Whitney rank test. (c) Correlation of serum IL-27 levels with circulating Th17 cells. Spearman's rank correlation test was used to assess the correlations. CD: Crohn's disease; CDR: CD patients in remission; CDA: CD patients in activity.

**Figure 6:**
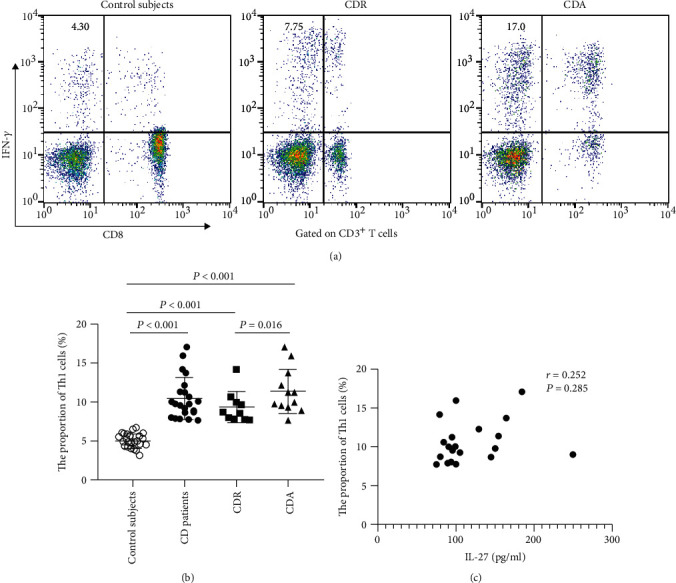
Circulating Th1 cells in healthy control subjects, all CD patients, CDR patients, and CDA patients and their relationship with serum IL-27 levels. (a) Representative dot plots of IFN-*γ* expression in peripheral CD4^+^ T cells (gate CD3^+^CD8^−^ T cells) of HC subjects, all CD patients, CDR, and CDA. (b) Scatter plot showing the proportion of circulating Th1 cells and the results of statistical analysis by using the nonparametric Mann–Whitney rank test. (c) Correlation of serum IL-27 levels with circulating Th1 cells. Spearman's rank correlation test was used to assess the correlations. CD: Crohn's disease; CDR: CD patients in remission; CDA: CD patients in activity.

Finally, we examined the correlation between IL-27 levels and the proportion of Th17 and Th1 cells in CD patients. As shown in Figures 5 and 6, we found that the proportion of Th17 and Th1 cells was significantly higher than that in control subjects (Th17 cells, median (IQR), CD vs. HC: 2.51 (2.04, 3.07) vs. 0.90 (0.70, 1.09) %, *P* < 0.001; Th1 cells, median (IQR), CD vs. HC: 9.72 (8.47, 11.54) vs. 4.94 (4.29, 5.81) %, *P* < 0.001). CD patients exhibited a significantly higher proportion of Th17 cells than those in remission (2.80 (2.15, 3.32) vs. 2.11 (2.04, 2.49) %, *P* = 0.015). As shown in Figure 6, the proportion of Th1 cells of CD patients in activity was higher than that of patients in remission (10.62 (9.28, 13.29) vs. 8.64 (7.88, 10.16) %, *P* = 0.016). Additionally, a significantly positive correlation existed between IL-27 levels and Th17 cells (*r* = 0.519, *P* = 0.013, Figure 5(c)). However, there were no significant associations between IL-27 levels and Th1 cells (*r* = 0.282, *P* = 0.285, Figure 6(c)). These data indicated that high serum IL-27 levels may play an important role in the differentiation of Th17 cells in CD patients, especially in the active stage.

## 4. Discussion

Our study revealed that the IL-27 levels were elevated in the serum samples of CD patients compared with control subjects and were especially higher in active CD patients. We also showed that IL-27 was positively correlated with CRP, ESR, CDAI, and SES-CD scores but negatively correlated with ALB and Hb. Additionally, the proportion of Th17 cells was closely related to IL-27 levels in the serum of CD patients. IL-27 combined with CRP could effectively predict disease activity (AUC4 = 0.88). Collectively, these results may indicate that IL-27 overproduction is positively correlated with the disease activity of CD patients and has a proinflammatory role in the pathogenesis of intestinal inflammation.

IL-6/IL-12 family cytokines play a vital role in regulating inflammatory responses [25]. Recently, several studies revealed that IL-27, a new member of the IL-6/IL-12 family of cytokines, plays a controversial role in regulating the immune system. A previous animal study showed that IL-27 is involved in the immunopathology of TNBS-induced colitis in rats via the TLR4/NF-*κ*B signaling pathway [26]. However, Hanson et al. [27] found that delivery of IL-27-productive bacteria can attenuate immune colitis in mice. Most studies have focused on the role of IL-27 on T cells, where receptor ligation results in activation of the Th1 transcription factors T-bet and STAT1 [28]. In the same inflammation animal models, IL-27 was proven to play a proinflammatory function. For example, IL-27 receptor-deficient mice were protected against proteoglycan-induced arthritis [29], and IL-27 knockout mice had lower Th1-related cytokine levels, lower anti-dsDNA antibody levels, and higher survival rates in lupus model mice [30]. Therefore, IL-27 may trigger inflammation in different diseases, including rheumatoid arthritis (RA) and inflammatory bowel disease (IBD). A previous study found that IL-27 was elevated in the serum of RA patients and could serve as a biomarker of disease activity in RA [31]. Furuzawa et al. confirmed that the tissue gene and protein expression of IL-27 in active IBD was significantly elevated and related to disease activity [21]. Consistent with previous studies, our study found that serum IL-27 levels in CD patients were significantly increased, and the levels in CDA were higher than those in CDR. Further correlation analysis confirmed that IL-27 was positively correlated with a variety of serum and fecal inflammatory markers and positively correlated with CDAI (clinical disease activity index) and SES-CD (endoscopic disease activity score) in CD patients. Our results indicated that IL-27 may be a potential biomarker to evaluate the disease activity of CD.

The abnormal activity of Th17 cells has an important role in the pathogenesis of CD [32, 33]. In accordance with previous studies, our data showed that the proportion of Th17 cells was increased in the peripheral blood of CD patients, particularly in active CD. In view of the pathogenicity of Th17 cells, it is particularly important to study how Th17 cells are regulated by human immune cells. Although there is abundant information about the differentiation of Th17 cells in mice, there is still little research on the differentiation pathway of Th17 cells in humans. However, the induction and regulation of Th17 cells are still controversial subjects. Some experimental studies confirmed that IL-27 could act as a cytokine to inhibit Th17 through several potential mechanisms [34, 35]. In contrast, another study revealed that IL-27 was elevated and could strongly induce IL-17 production in systemic sclerosis patients [36]. Interestingly, we found that elevated serum levels of IL-27 were significantly and positively correlated with the proportion of Th17 cells in CD patients. Most recently, one study reported that in liver injury patients, serum IL-27 was increased and positively related to Th17 cells [21], which also supports our findings. Collectively, our data provides new clinical evidence that IL-27 may be a positive regulator of the differentiation of Th17 cells but may also be committed to Th17 cells. Therefore, further experimental studies are needed to elucidate whether IL-27 regulates the development of Th17 cells and determine its role in the pathogenesis of CD.

There are several limitations to our study. First, we did not detect the levels of IL-27 protein in inflamed tissue and comprehensively evaluate the role of IL-27 in the development of the CD microenvironment. Second, due to the limited sample size, we did not analyze the serum IL-27 levels of CD patients with different disease activity severities. Finally, we did not further confirm the effect of serum IL-27 on Th17 cell differentiation in vitro. In the future, our team will further carry out relevant basic experiments to investigate the regulation of IL-27 on Th17 cells and its role in intestinal inflammation.

In summary, our study demonstrates that serum levels of IL-27 were significantly elevated in CD patients and positively correlated with several serum biomarkers and FC, as well as CDAI and SES-CD, and that IL-27 combined with CRP may be a potential biomarker for CD activity. IL-27 may participate in the pathogenesis of CD by regulating Th17 cell differentiation. These studies shed new insight into the dysregulation of the immune system and may provide new therapeutic targets for the treatment of CD in the future.

## Figures and Tables

**Table 1:** Clinical characteristics of the CD patients and control subjects.

	Crohn's disease (*n* = 60)	Control (*n* = 32)	*P*
Age (years)	27 (20.25, 31.50)	30 (23, 41.5)	0.160
Gender			
Female	13	12	0.140
Male	47	20	
Disease duration (months)	30.45 (3.20, 188.10)		
Age at diagnosis			
A1 (<16 years)	6	—	
A2 (16~40 years)	48	—	
A3 (>40 years)	6	—	
Disease location			
L1 (ileum)	21	—	
L2 (colon)	6	—	
L3 (ileum-colon)	33	—	
L4 (upper gastrointestinal tract)	2	—	
Disease behavior			
B1 (nonstenotic, nonpenetrating)	34	—	
B2 (stenotic)	22	—	
B3 (penetrating)	4	—	
p (perianal)	25	—	
Serum IL-27 level (pg/ml)	110.0 (95.0, 145.0)	85.0 (80.0, 95.0)	<0.001
Serum IL-23 level (pg/ml)	23.7 (16.2, 35.9)	20.6 (14.4, 23.6)	0.162
Medical therapy			
Mesalazine only	14		
Steroids±mesalazine	14		
Azathioprine±mesalazine	8		
Infliximab±mesalazine	35		

Values are expressed as median (interquartile range (IQR)).

## Data Availability

The original data supporting this research conclusion will be available from the corresponding author.
